# *Lucilia cuprina* genome unlocks parasitic fly biology to underpin future interventions

**DOI:** 10.1038/ncomms8344

**Published:** 2015-06-25

**Authors:** Clare A. Anstead, Pasi K. Korhonen, Neil D. Young, Ross S. Hall, Aaron R. Jex, Shwetha C. Murali, Daniel S.T. Hughes, Siu F. Lee, Trent Perry, Andreas J. Stroehlein, Brendan R.E. Ansell, Bert Breugelmans, Andreas Hofmann, Jiaxin Qu, Shannon Dugan, Sandra L. Lee, Hsu Chao, Huyen Dinh, Yi Han, Harsha V. Doddapaneni, Kim C. Worley, Donna M. Muzny, Panagiotis Ioannidis, Robert M. Waterhouse, Evgeny M. Zdobnov, Peter J. James, Neil H. Bagnall, Andrew C. Kotze, Richard A. Gibbs, Stephen Richards, Philip Batterham, Robin B. Gasser

**Affiliations:** 1 Faculty of Veterinary and Agricultural Sciences, The University of Melbourne, Parkville, Victoria 3010, Australia; 2 Department of Human and Molecular Genetics, Baylor College of Medicine, Houston, Texas 77030, USA; 3 School of Biosciences, The University of Melbourne, Parkville, Victoria 3010, Australia; 4 Structural Chemistry Program, Eskitis Institute for Drug Discovery, Griffith University, Brisbane, Queensland 4111, Australia; 5 Department of Genetic Medicine and Development, University of Geneva & Swiss Institute of Bioinformatics, CH-1211 Geneva, Switzerland; 6 Ecosciences Precinct, Queensland Alliance for Agriculture and Food Innovation (QAAFI), Queensland Bioscience Precinct, The University of Queensland, St Lucia, Brisbane, Queensland 4072, Australia; 7 CSIRO Agriculture Flagship, Queensland Bioscience Precinct, St Lucia, Brisbane, Queensland 4067, Australia

## Abstract

*Lucilia cuprina* is a parasitic fly of major economic importance worldwide. Larvae of this fly invade their animal host, feed on tissues and excretions and progressively cause severe skin disease (myiasis). Here we report the sequence and annotation of the 458-megabase draft genome of *Lucilia cuprina*. Analyses of this genome and the 14,544 predicted protein-encoding genes provide unique insights into the fly's molecular biology, interactions with the host animal and insecticide resistance. These insights have broad implications for designing new methods for the prevention and control of myiasis.

## Abstract (web summary)

*Lucilia cuprina* is a parasitic blowfly of major economic importance worldwide that feeds on the tissues of animals such as sheep. Here, the authors sequence the genome of *L. cuprina* and provide insights into the fly's molecular biology, interactions with the host animal and insecticide resistance.

## Graphical abstract

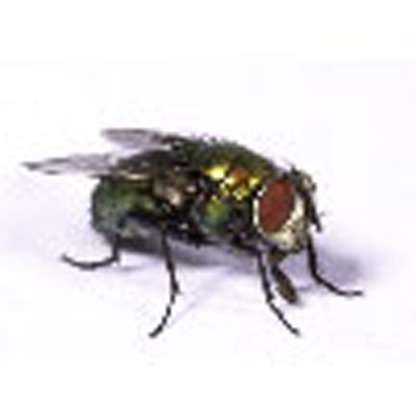

Insect vectors that transmit viral, bacterial and/or parasitic diseases are of major socioeconomic importance globally^1^. For instance, some dipteran flies are primary parasites of plants or animals^1^,^2^, and can also act as vectors of pathogens^3^. In particular, some blowflies, such as *Lucilia* spp., are parasitic and feed on the tissues of animals, such as sheep^4^. The disease caused by blowfly (flystrike or myiasis) is a serious problem in many countries around the world^2^; in Australasia alone, hundreds of millions of dollars are lost annually due to reduced wool and body growth in sheep^4^ as well as costs associated with blowfly treatment/control and animal morbidity^4^. The principal fly involved in flystrike is *Lucilia cuprina* (Insecta, Diptera, Calliphoridae), with the majority of myiasis cases being initiated by this species^4^,^5^.

Adult *L. cuprina* females are attracted to odours from the host, particularly those associated with bacterial infections in damp fleece, or areas of fleece or skin soiled by urine or faeces^5^. They lay eggs (∼200 eggs per batch per female fly) on skin areas of high humidity^5^. Larvae (maggots) hatch from eggs within 8 h to 3 days and proceed through three stages of development^5^. They use their mouth hooks to abrade the skin and feed on skin secretions, dermal tissues and blood^5^. The resultant damage or ‘strike' is mainly due to mechanical and chemical effects of larval feeding as well as protease release, which can cause severe disease and, in extreme cases, death^4^.

Although blowfly strike has been the subject of extensive investigations over many years, and some control methods have been developed, an effective and permanent solution to flystrike has not yet been found. A common means of prevention is mulesing^6^, a surgical procedure that removes wool-bearing skin from around the tail and from either side of the breech area of sheep, resulting in an area devoid of wrinkles or skin folds, reducing the accumulation of secretions that attract flies. This controversial practice is heavily scrutinized by animal welfare organizations, because of physical, behavioural and psychological indicators of stress that result from mulesing^7^. Therefore, there is a need for an alternative to this surgical practice. Although immunogens have been studied^8^, no effective vaccine is yet available against blowfly^4^. Insecticides continue to be heavily relied upon to prevent and treat flystrike; however, this reliance is becoming increasingly problematic due to chemical residue problems in animal products and the rapid emergence of resistance in blowflies against many classes of insecticides^4^. Profound insights into the fundamental, molecular processes in this fly could provide a sound basis for the design of new interventions (for example, vaccines or insecticides). To underpin these areas, and as part of the 5000 Insect Genome (i5k) Project^9^, we sequenced and characterized the 458-megabase (Mb) draft genome of *L. cuprina* and defined the global molecular landscape of this fly. We also investigated particular genes involved in insecticide resistance, expressed a *L. cuprina* nicotinic acetylcholine receptor (nAChR) subunit (*Lc*α6) gene in *Drosophila melanogaster* and assessed this subunit's capacity to rescue spinosad resistance in *D. melanogaster* mutants. The present genomic resource for a parasitic fly of major agricultural importance provides a solid foundation for exploring the molecular basis of blowfly development and reproduction, fly–host interactions, the pathogenesis of myiasis and, importantly, insecticide resistance.

## Results

### Genome assembly and repeat content

We sequenced the genome of *L. cuprina* at ∼100-fold coverage (Table 1 and Supplementary Data 1), producing a final draft assembly of 458 Mb (scaffold N50: 744,413 bp; Table 1), with a mean GC content of 29.3%. This genome is more than twice the size of that of *D. melanogaster* (180 Mb), larger than that of *Glossina morsitans* (366 Mb) and smaller than that of *Musca domestica* (691 Mb)^10^,^11^,^12^. We detected 96.0% complete and 100% partial 248 core essential genes by CEGMA, indicating that the assembly represents a substantial proportion of the entire genome. The estimated repeat content of this draft genome is 57.8% (265 Mb), comprising 2.7% DNA transposons, 4.6% retrotransposons, 16.7% unclassified dispersed elements and 5.2% simple repeats (Supplementary Data 2). We identified 78,741 distinct retrotransposons representing at least three categories (16,688 LTRs, 61,619 LINEs and 434 SINEs), with *ERV_classII* predominating for LTRs (*n*=423) and *L3/CR1* for non-LTRs (*n*=6,358). We also identified 60,359 DNA transposons, of which *hAT-Charlie* (*n*=490) and *TcMar-Tigger* (*n*=410) predominated (Supplementary Data 2).

### Gene set and functional annotation

**Figure 1:**
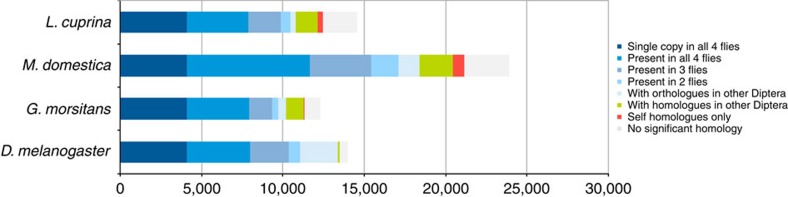
Orthology comparison among *Lucilia cuprina*, *Drosophila melanogaster*, *Glossina morsitans* and *Musca domestica*. A total of 4,106 genes are SCOs that are shared among the four fly species; 12,160 *L. cuprina* genes are shared with at least one other species of dipteran. In this comparison, 2,062 genes (14.2%) are unique to *L. cuprina*.

We predicted 14,554 coding genes using *de novo* and homology-based predictions, of which 10,121 were supported by mapping RNA-seq reads (*n* ≥5) derived from larval stages (mixed) and adults (both sexes) of *L. cuprina*. Mean gene, exon and intron lengths were 12,197, 432 and 2,560 bp, respectively, with an average of 4.5 exons per gene (Table 1), similar to the findings for the genomes of *D. melanogaster*, *G. morsitans* and *M. domestica*^10^,^11^,^12^. A total of 4,106 genes are single-copy orthologues (SCOs) shared among the four fly species, and 12,160 genes are shared with at least one other species of Diptera (Fig. 1). In contrast, 2,062 genes (14.2%) are unique to *L. cuprina*, with no homologues detected in any other dipteran for which genome sequence data are currently available (Fig. 1). Of the entire *L. cuprina* gene set, 9,822 genes (67.5%) have an orthologue (E-value cutoff ≤10^−5^) linked to one or more of 254 known biological (KEGG) pathways, most of which mapped to those in *D. melanogaster* (see Supplementary Data 3). The completeness of the genome is further supported by the CEGMA results (Supplementary Data 1). By inference, the majority of the *L. cuprina* gene set is represented in the present genomic assembly, and supported by extensive transcriptomic and inferred proteomic data (*n*=10,121 and 11,553 molecules, respectively) from multiple public databases.

Of the 14,554 protein-encoding genes of *L. cuprina*, 12,160 (83.6%) had homologues in other dipterans; 10,396 (71.5%), 9,023 (62%) and 7,659 (52.7%) had significant matches in the InterProScan, UniProtKB/Swiss-Prot and KEGG BRITE databases, respectively. Using all other accessible protein- and/or conserved protein domain-databases, we annotated 12,160 (83.6%) of the protein-coding genes of *L. cuprina* (Table 2). A genome-wide analysis revealed higher numbers of SCOs shared between *L. cuprina* and *G. morsitans* (*n*=6,183) and between *L. cuprina* and *M. domestica* (*n*=5,769) than between *L. cuprina* and *D. melanogaster* (*n*=3,222). A syntenic comparison using these SCOs within three scaffolds (nos. 18, 23 and 42) of >3.5 million bp each in the *L. cuprina* genome revealed small numbers of scaffolds with a high degree of synteny and sharing blocks of 5 or more SCOs in the genomes of *D. melanogaster* (*n*=2; scaffolds nos. 23 and 42), *G. morsitans* (*n*=5; scaffolds nos. 18, 23 and 42) and *M. domestica* (*n*=6; scaffold nos. 23 and 42; Supplementary Figs 1–3). The largest block of SCOs (*n*=15) was found between *L. cuprina* (scaffold no. 42) and *M. domestica* (MdScaffold18918), with the second largest block (*n*=13) found between *L. cuprina* (scaffold no. 18) and *G. morsitans* (scaf7180000643747). These results are consistent with current knowledge of the evolutionary relationships of dipterans and the taxonomic placement of *D. melanogaster* within the Acalyptratae as well as *L. cuprina*, *G. morsitans* and *M. domestica* within the Calyptratae.

### Enzymes, channels, pores and transporters

In total, we identified 260 peptidases representing the six main groups (that is, metallo-, cysteine, serine, aspartic, threonine peptidases and some of an unknown catalytic type), with the serine (*n*=96; 36.9%), metallo- (*n*=91; 35.0%) and cysteine (*n*=46; 17.7%) peptidases predominating (Supplementary Data 4). Most abundant are S1 chymotrypsin (*n*=74), S28 (*n*=4) and S9 α/β hydrolases, including prolyl oligopeptidase (*n*=4), among the serine peptidases; M13 neprilysin (*n*=19), M12 astacin/adamalysin (*n*=17) and M14 carboxypeptidase A (*n*=12) among the metallo-peptidases; and the C1 papain (*n*=8), C14 caspase (*n*=7) and C19 ubiquitin-specific peptidase (*n*=7) families among the cysteine peptidases. Similar peptidase groups, including families S1, S9, M14, C19 and M13, are represented in the *Glossina* genome^11^. Interestingly, 254 (97.7%) of the 260 peptidases identified in *L. cuprina* have homologues in the tsetse fly.

We identified at least 167 protein kinases and 199 phosphatases to be encoded in the *L. cuprina* genome (Supplementary Data 5 and 6). The kinome includes serine/threonine (87.4%) and tyrosine (12.6%) protein kinases. The phosphatome includes principally protein serine/threonine (81.8%) and protein tyrosine (10.1%) phosphatases as well as a small number of haloacid dehalogenase phosphatases (8.1%). In addition, we predicted at least 92 GTPases to be encoded in *L. cuprina*, including 11 large (heterotrimeric) and 81 small (monomeric) G-proteins representing the Rab (*n*=32), Arf/Sar (*n*=16), Ras (*n*=21), Rho (*n*=7) and Ran (*n*=3) families as well as some unclassified molecules (Supplementary Data 7). Many of these GTPases, including Ras and Rho, likely coordinate the signal transduction pathways associated with organogenesis and morphogenesis (cell division and differentiation) in the fly. For example, these molecules are involved in the dynamic assembly, disassembly and reorganization of the actin and microtubule cytoskeletons, the interaction of growing axons with other cells and extracellular matrices, the delivery of proteins and lipids to axons through exocytic machinery and/or the internalization of proteins or membranes at the leading edge of the growth cone via endocytosis^13^. Examples of dominant small GTPase homologues are Ras64B, Rab23, Gαf, Arl1, Arl2, Rab6, RabX1 and Ras85D whose *D. melanogaster* orthologues are essential for larval growth and/or development (www.flybase.org). Therefore, we propose that some of these and related enzymes are potential targets for interventions against *L. cuprina* based on their roles in other organisms such as *Drosophila*^14^,^15^.

In this context, the large complement of receptor, channel, pore and transporter proteins in *L. cuprina* is also of particular interest, considering that many common insecticides target some of these proteins^16^,^17^. We predicted 197 G protein-coupled receptors (GPCRs) to be encoded in *L. cuprina*, including rhodopsins (*n*=73), secretin receptors (*n*=18), metabotropic glutamate receptors (*n*=9) and some unclassified proteins (Supplementary Data 8). We also predicted 136 ion channel proteins (Supplementary Data 9), the majority of which represent the voltage-gated cation channel superfamily (*n*=31), such as the potassium (61.3%) and the calcium (35.5%) channel families, and the epithelial and related channel superfamily (*n*=28) including acid-sensing ion channels. We also found channels of the cys-loop superfamily (*n*=24), some of which (for example, nAChRs) are recognized targets of several insecticides in *L. cuprina*^18^. Molecules involved in chemoreception (*n*=93), including a number of gustatory and odorant receptors, were relatively abundant, some of which (for example, *Gr63a*) are likely involved in the detection of host carbon dioxide^19^, and might represent intervention target candidates. In addition, 367 transporters were inferred for *L. cuprina* (Supplementary Data 10), including an abundance of proteins of the solute carrier family (46.4%), major facilitator superfamily (24.3%) and ABC transporters (*n*=42), some of which have been shown to relate to insecticide resistance via the active transport of drugs out of cells^17^,^20^. We also identified seven aquaporin (*aqp*) genes that likely facilitate rapid, highly selective water transport into and out of cells, thus regulating osmotic pressure in cells. On the basis of evidence from other flies^21^, these aquaporins are proposed to play a role in the hydration of saliva during feeding, the reduction in volume of ingesta for the purpose of efficient digestion, the mobilization of water to progeny during oogeny and to cold and heat tolerance in *L. cuprina.*

### Comparative transcriptomic analyses

To explore the molecular biology of *L. cuprina*, we compared transcription between male and female adults, and between adults and mixed larval stages. Transcripts in female and male adults were highly enriched (*n*=86 and 138, respectively) for gene ontology annotations such as oogenesis and vitelline membrane formation in the females, and sensory perception of chemical stimuli and defence response in the males (Supplementary Data 11 and 12).

The male-enriched transcript set (Supplementary Data 12) represents genes encoding testis-specific serine kinases (proposed to be involved in DNA condensation during post-meiotic chromatin remodelling) as well as three Niemann–Pick type C2 proteins, which are believed to regulate sterol homeostasis and the biosynthesis of 20-hydroxyecdysone, a steroidal insect moulting hormone of *Drosophila*^22^. Niemann–Pick type C2 proteins might play a central role in chemical communication in *L. cuprina*, based on evidence for *Camponotus japonicas* (Japanese carpenter ant)^23^. A total of 15 proteins belonging to the sperm-coating protein-like extracellular (SCP/TAPS) protein family were identified based on their characteristic CAP domain (IPR014044). Most SCP/TAPS proteins characterized to date are often secreted and function extracellularly in a variety of physiological processes, such as fertilization or immune responses^24^,^25^. For instance, in *Drosophila*, 26 SCP/TAPS genes have been identified, with 70% preferentially expressed in males^26^, some of which are likely involved in male-specific reproductive processes. Further investigation of these genes and their function is warranted, as SCP/TAPS proteins of helminths can play key roles in reproduction, immunomodulation and/or host invasion^25^, and might thus represent potential insecticide or vaccine candidates for various ecdysozoans including blowfly. Proteins phormicin (a defensin)^27^ and cecropin C^28^, two antimicrobial peptides of the haemolymph, known to be involved in cell-free immune attack of insects mainly against Gram-positive and/or -negative bacteria, were also represented in the male-enriched transcript set. The crucial role of these two peptides appears to link with a transcription level that is among the highest of any gene and stage of *L. cuprina* (Supplementary Data 12); the extent of male-enriched transcription likely reflects an extensive defence arsenal required to protect male flies from the onslaught of a wide range of microbes of different classes subsisting on diverse food sources/diets (including nectar, honeydew and/or carrion)^29^.

Among the female-enriched transcripts are various orthologues associated with reproductive processes, including oogenesis/egg laying and eggshell formation (for example, *Vm26Aa*, *Vm34Ca*, *Vm32E*, *del* and yolk protein (*yp*) genes; see, for example, FlyBase) and/or female sex-determination (for example, *stil*) (see, for example, FlyBase), all of which have orthologues in *Drosophila* spp. (Supplementary Data 11). While the vitelline membrane (*Vm*) genes encode proteins of the first layer of the eggshell produced by the follicular epithelium, the lipase-derived yolk proteins are required for vitellogenesis in *L. cuprina*^30^. The four *yp* genes specific to the female blowfly compare with three (*yp1*, *yp2* and *yp3*) in *Drosophila*, but only one in *Glossina*^11^; this difference in the number of orthologues is hypothesized to relate to oviparous reproduction in the two dipterans^31^ *vis-à-vis* adenotrophic viviparity in the glossinid fly^30^. By contrast, transcripts enriched in mixed-stage larvae (*n*=256) of *L. cuprina* including those encoding enzymes (for example, cathepsin-D and chymotrypsin) involved in digestion, peritrophin-44 and various proteins linked to growth and development (including *Ccp84Ab*, *Lcp1*, *Lcp2*, *Lcp65Ab1* and *Edg84A*) were prominent (Supplementary Data 13). The cluster of genes (*Lcp1*, *Lcp2* and *Lcp65Ab1*) encoding cuticle proteins is integral to determining characteristics of the cuticle^32^, and orthologue *Edg84A* likely governs *L. cuprina* metamorphosis, being regulated through transcription factors (TFs) homologous to FTZ-F1 and DHR3 of *D. melanogaster*^33^,^34^,^35^. Interestingly, substantial transcription of the peritrophin-44 gene in larvae relative to adults is consistent with an abundance of this protein in the peritrophic membrane of all three larval instars, but trace amounts in adult *L. cuprina*^36^. Through its binding to chitin, peritrophin-44 likely maintains the structure and porosity of the peritrophic membrane, a semi-permeable chitinous matrix lining the gut, which is proposed to have key roles in maintaining gut structure, protection from microbial invasion and/or the facilitation of digestion, possibly together with cathepsin-D and/or chymotrypsin.

Interestingly, 15% of the 480 transcripts enriched in larvae or either gender of the adult stage had no homologue in any other organism for which the data are currently available in public databases. Most of the 70 orphan (that is, unannotated) transcripts were identified in mixed larvae (*n*=37) compared with male (*n*=27) and female (*n*=6) adults. These findings are consistent with those for other dipterans such as *Glossina* and *Musca*, which have similar complements of orphan genes^11^,^12^; in a conservative comparison of 28 insect species, similar numbers of orphan genes for individual species were reported^37^. The presence of a considerable number of orphan genes emphasizes the uniqueness of the biology of *L. cuprina* and encourages in-depth studies of the expression and functions of these unique molecules throughout the fly's life cycle. Some of them are likely involved specifically in host invasion and/or interactions, and might represent highly selective insecticide or vaccine targets.

### Parasite–host interactions and potential vaccine molecules

Excretory/secretory (ES) proteins can also play critical roles in the immunobiological relationship between *L. cuprina* larvae and the host animal^8^. Here we predicted the secretome of *L. cuprina* to include 1,004 proteins with a diverse array of inferred functions, of which 234 had homologues in two or more public databases (see Supplementary Data 14). Conspicuous were orthologues encoding 58 peptidases, including 47 serine proteases (for example, chymotrypsin and trypsin) and 11 aspartic proteases (for example, cathepsin). In addition, 25 genes encoding hydrolases (for example, chitinase and lipoprotein lipase), 12 mucin-like proteins, seven peritrophin proteins, seven peptidase inhibitors, including serpin B, and 30 cuticle-like proteins as well as 194 orphan molecules were identified. Many secreted peptidases representing the ‘degradome' (and their respective inhibitors) have central roles in larval establishment, degradation of blood, skin and various proteins and/or the activation of inflammation and immune responses^4^,^38^; some of these peptidases could represent intervention targets in the larval stage of *L. cuprina*. Of the genes encoding the 1,004 predicted ES proteins, 852 were transcribed in larval stages, and 79 were exclusive to these stages. On the basis of comparison with other ecdysozoans, 79 of the 852 (9.3%) ES molecules are predicted to be involved in host interactions and/or are immunogenic (see Supplementary Data 14), and include 11 cuticular proteins, 2 serine peptidases and peritrophin-44. Some of the annotated molecules, such as peritrophins, have already been shown to regulate larval growth and survival^39^ and induce temporary, protective immunity in experimental sheep against challenge infection with *L. cuprina*^40^. Overall, the present genomic and transcriptomic data sets infer that *L. cuprina* has a major arsenal of ES proteins, including some orphan molecules, which are likely involved in inducing and/or modulating immune responses in the host animal. A detailed understanding of the roles of these molecules could contribute towards developing subunit vaccines against flystrike^8^.

### Insecticide-resistance genes and functional analysis of *Lc*α6

**Figure 2:**
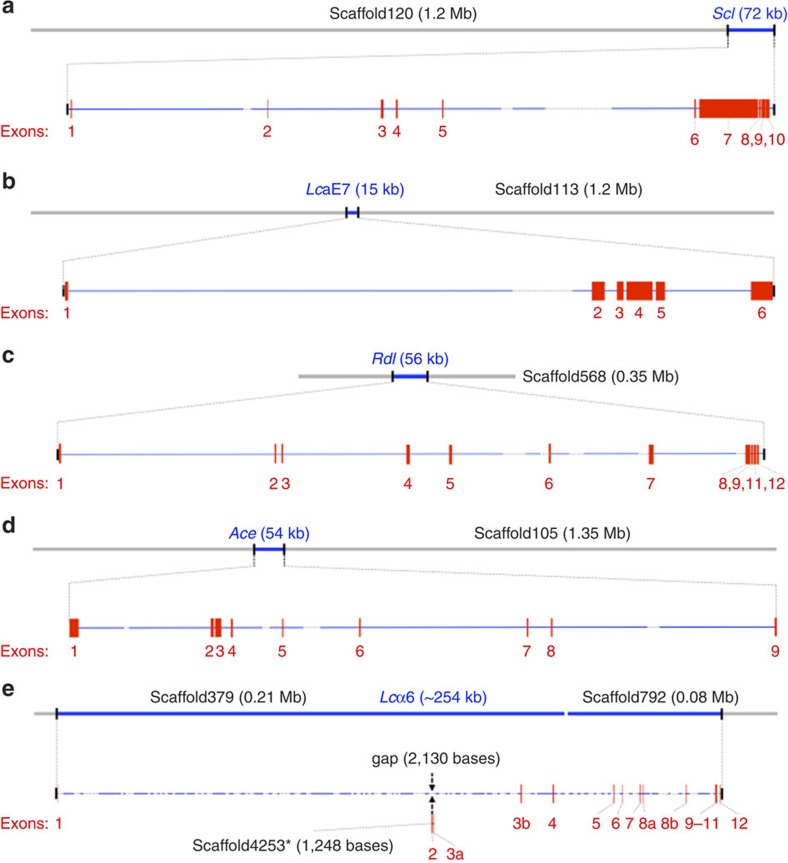
Structures of five insecticide-resistance genes in *Lucilia cuprina*. Diagrams show the genomic structures of *L. cuprina* genes, which have been implicated in resistance to insecticides used to control *L. cuprina* — *Scl* (encoding a transmembrane receptor important for intracellular signalling and proposed to modify phenotypes associated with organophosphorus (OP) insecticide resistance conferred by *Rop1*), *Lc*aE7 (=*Rop1*, encoding carboxylesterase E3; associated with OP insecticide resistance), *Rdl* (resistance to dieldrin), *Ace* (acetylcholinesterase) and *Lc*α6 (nAChR α6 subunit). Genes *Scl*, *Lca*E7, *Rdl* and *Ace* were located to scaffolds nos. 120, 113, 568 and 105, respectively (**a**–**d**). It was also noted that the current *L. cuprina* assembly did not contain *Rdl* exon 10 compared with the existing *Lucilia* cDNA sequence (GI: 2565319) (**c**). The absence of *Rdl* exon 10 is supported by RNA-seq data. The *Lc*α6 gene (254 kb) was represented on scaffold nos. 379, 4,253 and 792 (**e**), and contains 10 exons including four *L. cuprina*-specific α6 exons (called 3a, 3b, 8a and 8b; all transcribed). The *Lc*α6 gene is located in a highly repetitive region, and manual sequence analysis of the paired-end reads successfully mapped the scaffold4253 (containing *Lc*α6 exons 2 and 3b) to a 2.1 kb gap within scaffold379. Gene regions are indicated by blue lines; gaps within gene regions are depicted as dashed lines. Red vertical lines/boxes represent exons.

Although there is little detailed knowledge of the molecular basis of insecticide resistance in *L. cuprina*, numerous studies^4^ have inferred or proposed a direct or indirect involvement of various genes in such resistance, for both metabolic and target site insensitivity-resistance mechanisms. We have annotated genomic loci for five genes associated with particular resistances, including *Ace* (acetylcholinesterase, the target for organophosphorus insecticides, OPs), *Rdl* (resistance to dieldrin), *Lc*aE7 (or *Rop1*—resistance to OPs; encodes carboxylesterase E3), *Scl* (transmembrane receptor for intracellular signalling, proposed to be modifier of phenotypes associated with *Rop1*-mediated OP resistance) and *Lc*α6 (nAChR α6 subunit) (Fig. 2). Importantly, previously, we had characterized full-length *L. cuprina* complementary DNA (cDNA) sequences, which assisted direct cDNA–gDNA alignments to support the definition of exon–intron boundaries in the present study. Using the genomic and transcriptomic data sets for *L. cuprina*, we identified these genes in long genomic scaffolds and established their structures (Fig. 2), which should provide a foundation for functional studies of insecticide resistance in *L. cuprina* and other pests.

From previous studies^41^,^42^,^43^, we know that resistance to the widely used insecticide spinosad is due to loss-of-function (LOF) mutations in the gene encoding the nAChR α6-like subunit. Mutations in α6-like receptors in *D. melanogaster*, *Plutella xylostella* and *Frankliniella occidentalis* led to high levels of spinosad resistance, which suggests a common mechanism across insect species^41^,^42^,^43^. The model insect *D. melanogaster* proved to be very useful to explore this aspect. LOF mutations in the *D. melanogaster* orthologue of this gene (*D*α6) confer high levels of resistance, suggesting that spinosad exerts its lethal effect by binding to this subunit. Introducing a *D*α6 orthologue from various insect pest species into this LOF background has been shown to render *D. melanogaster* susceptible to spinosad, indicating that the introduced receptor subunit is functional and binds spinosad when expressed in *D. melanogaster*^44^. Therefore, we proposed that α6 LOF mutations confer high-level resistance to spinosad in various insect pests.

**Figure 3:**
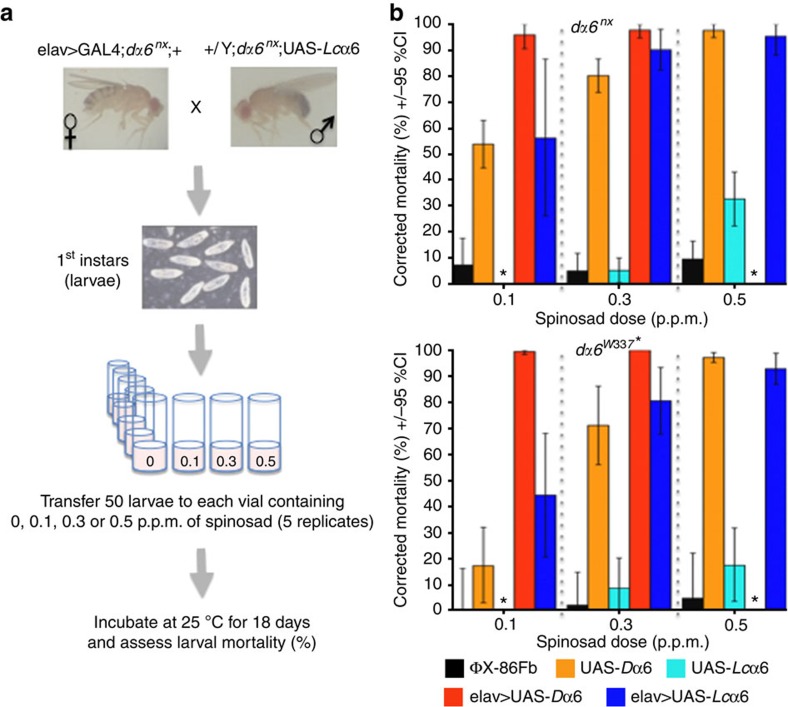
Experimental design for the expression of the *L. cuprina* nAChR subunit gene (*Lc*α6) in *Drosophila melanogaster* and rescue of spinosad resistance in *D. melanogaster* (*D*α6) mutants. (**a**) Virgin female elav>GAL4;*dα6*^*nx*^ flies were crossed with male *dα6*^*nx*^;UAS-*Lc*α6 flies. The elav-driver produces GAL4 in neuronal cells, and the GAL4 binds to the UAS site to express the *Lc*α6 subunit that can be assembled into nAChRs. All individuals of the F1 generation have copies of the driver and the construct. First instar larvae were placed in sets of 50 on culture medium containing 0.1, 0.3 and 0.5 p.p.m. of spinosad, and allowed to develop. Mortality (%) was recorded on day 18 and normalized against control mortality. (**b**) Results from rescue experiments using the *D. melanogaster* GAL4:UAS system in different *D*α6 mutant allele backgrounds: *Lc*α6–rescue showed no significant mortality in the *D. melanogaster* line ΦX-86Fb^46^ (Black) at 0.1, 0.3 and 0.5 p.p.m. of spinosad in a *dα6*^W337*^ background and low mortality (9.4%±6.8) was seen only at 0.5 p.p.m. but not at the two lower doses in a *dα6*^*nx*^ background. The UAS-*D*α6 insertion line (Light orange) was susceptible to spinosad at all three doses, whereas the UAS-*Lc*α6 line (Cyan) was susceptible only at 0.5 p.p.m. (0.1 p.p.m. not tested). The driver line elav>GAL4 expressing *D*α6 was highly susceptible at all three doses (Dark orange). The expression cross for the *D. melanogaster* α6 subunit had high mortality (>90%) on medium containing 0.1 and 0.3 p.p.m. spinosad (0.5 p.p.m. not tested), while the *L. cuprina* α6 subunit expression cross (Dark blue) showed significant, increasing spinosad susceptibility, with >90% mortality at 0.5 p.p.m. The bars represent 95% confidence intervals (CIs) calculated using a modified Abbott's correction^69^; five samples at 50 individuals each were tested for each dose. *—Not tested at that dose.

To examine whether α6*-*based spinosad resistance might evolve in *L. cuprina*, we performed heterologous expression of *Lc*α6 in *D. melanogaster* (Table 3), and assayed for functional rescue and insecticide susceptibility in transgenic flies. Utilizing the *D. melanogaster* GAL4:UAS system^45^, we cloned *Lc*α6 into either a *dα6*^*nx*^ or a *dα6*^W337*^ spinosad-resistant background (61- and 1,176-fold)^44^ and expressed *Lc*α6 in the elav>GAL4 driver line of *D. melanogaster* (Fig. 3). Rescue experiments showed that *Lc*α6 restored spinosad susceptibility in *D. melanogaster* (Fig. 3); no significant mortality in the *D. melanogaster* line ΦX-86Fb^46^ was observed using 0.1, 0.3 and 0.5 p.p.m. of spinosad in a *dα6*^W337*^ background, and low mortality (9.4%±6.8) was seen only at 0.5 p.p.m., but not at the two lower doses in a *dα6*^*nx*^ background. The UAS-*D*α6 insertion line was susceptible to spinosad at all three doses, whereas the UAS-*Lc*α6 line was susceptible only at 0.5 p.p.m. (due to ‘leaky expression' at the attP landing site^47^. The driver line elav>GAL4 expressing *D*α6 was highly susceptible at all three doses. Although transgenics with the *Lc*α6 subunit responded significantly at all doses, mortality at 0.1 p.p.m. was significantly lower than *D*α6 in both the backgrounds (*dα6*^*nx*^ and *dα6*^W337*^) when driven by elav>GAL4, showing that rescue was not as efficient as for *D*α6.

### Prospects for new insecticides

Clearly, the excessive use of various chemicals against *L. cuprina* has led to major insecticide-resistance problems^4^. Unfortunately, limited progress has been made in discovering new classes of insecticides effective against this parasite^4^. Genomic-guided drug target or drug discovery provides a promising approach to support screening and repurposing^48^; the goal of such discovery is to identify genes or gene products whose inactivation by one or more insecticides selectively kill fly larvae but do not harm the host animal. As gene-specific perturbation by double-stranded RNA interference is not yet practical for the direct evaluation of gene functions on a genome-wide scale in *L. cuprina*, gene essentiality can be predicted from functional genomic data (for example, lethality) for *D. melanogaster*, and this approach has already yielded credible insecticidal targets and provided insight into the mechanisms of resistance^48^. In *L. cuprina*, we inferred 988 genes with essential homologues/orthologues in *D. melanogaster* linked to lethal or semi-lethal phenotypes on gene silencing (Supplementary Data 15). We assigned highest priority to insecticide or vaccine target candidates inferred to be encoded by single genes, reasoning that lower allelic variability in *L. cuprina* populations would less likely give rise to resistance. We predicted 251 druggable genes/proteins using ChEMBL, of which 79 had interacting ligands that satisfy the Lipinski rule-of-three and rule-of-five, and are considered ‘MedChem-friendly' (Supplementary Data 16); one of them (*Rpd3*) is linked to lethal phenotypes in *D. melanogaster* (Supplementary Data 15). Conspicuous among the 79 druggable molecules are seven transporters and four ion channels that could represent primary targets for multiple classes of natural or synthetic insecticidal compounds. Other candidates among the 79 druggable proteins include 19 kinases, five peptidases, five growth factor receptors and seven TFs, some of which have been suggested as targets for proteinase inhibitors^49^, genetically modified baculoviruses^50^ or *Bacillus thuringiensis* endotoxins^51^.

Interestingly, in *L. cuprina*, we identified an SCO of *ladybird late* (*lbl*), a homeobox-containing gene encoding a TF that plays an essential role in regulating developmental processes, such as embryonic neurogenesis, myogenesis and/or cardiogenesis in *D. melanogaster*^52^. The sequence of *lbl* is relatively conserved due to its crucial regulatory functions in invertebrates and vertebrates^52^,^53^; we propose that *Lc-lbl* plays a key role in regulating the expression of reporter gene products in the adult female accessory gland of *L. cuprina*, as reported for *Drosophila*^52^. Given that female accessory glands perform essential reproductive functions (for example, fertilization and egg hatching), we believe that *Lc-lbl* could be critical for successful reproduction, which is consistent with evidence for some other insects, such as *Drosophila* and *Glossina*^54^,^55^. Gene sequence conservation among (some) insects and evidence of serious phenotypes (for example, reduced larval growth or abortion) on gene perturbation in selected dipterans^53^,^55^ indicate that this TF gene should be an important focus for comparative functional genomic explorations of developmental processes in both embryonic and adult female *L. cuprina*, and might serve as an intervention target in this fly.

## Discussion

The present genomic and transcriptomic exploration provides a global insight into the molecular biology of *L. cuprina.* We have elucidated molecules likely involved in host–fly interactions and immune responses, and studied transcriptional differences between stages and/or sexes of this parasitic fly. Over the years, there has been a major emphasis on the development of various control strategies to combat the blowfly, including mulesing, experimental vaccines, genetic transformation technologies and effective insecticides^4^. Although the use of insecticides against the blowfly has been successful, resistance in this insect has emerged to almost all currently used compounds.

The present investigation shows, for the first time, the structures of five genes related to resistance. For example, the *Lc*α6 gene is relatively large and complex, as in *D. melanogaster*, and spans several scaffolds in the draft genome of *L. cuprina*. The genomic sequences match well with the previously cloned *Lc*α6, including all of its alternative exons. Several features of this gene from other species, such as alternative splicing and RNA editing, are also conserved between *L. cuprina* and *D. melanogaster*^56^.

Susceptibility to spinosad was restored in transgenic *D. melanogaster (d*α6 mutant backgrounds) expressing the *Lc*α6 subunit. This finding shows functional conservation for this subunit and that *D. melanogaster* can serve as a useful model for the analysis of receptor function from other organisms such as *L. cuprina*. Despite the substantial difference in codon usage between the two species, and the differences in the chaperones and structural proteins required to fold, traffic and assemble a functional nAChR pentamer, the homologous subunit from *L. cuprina* is able to respond to spinosad in a manner quite analogous to that of *D. melanogaster*. This finding is concordant with those from a previous study^44^ that showed that several α6 subunits from other insect species (*M. domestica*, *Plutella xylostella* and *Bovicola ovis*) could also restore susceptibility to spinosad. Overall, these findings suggest that α6*-*linked resistance evolves in insect pests and emphasize a need to monitor such resistance. Clearly, the genomic and transcriptomic data sets for *L. cuprina* provide an important resource for exploring the biological functions of genes linked to insecticide resistance in parasitic flies.

To manage and prevent resistance, there continues to be a need for new insecticidal therapies and/or an effective vaccine to control flystrike. There is a major demand for a subunit vaccine based on ‘natural' or ‘hidden' antigens^5^ from larval stages, to induce an early, protective immune response in the host animal. From a fundamental viewpoint, knowing the global molecular biology of *L. cuprina* will now facilitate explorations of many aspects of the developmental and reproductive biology, physiology and biochemistry of *L. cuprina* as well as parasite–host interactions and the pathogenesis of myiasis. Recent technological advances also provide major prospects for systems biology investigations of the proteome and metabolome of *L. cuprina*. The present genome and transcriptomic data provide a solid foundation for the transition from ‘single-molecule' research to global molecular discovery in *L. cuprina*, and should accelerate post genomic explorations. This exciting prospect is likely to lead to a paradigm shift in our understanding of this enigmatic, parasitic fly and to significant advances in applied areas, including the development of new interventions through the investigation of essential, fly-specific molecules using functional genomic tools. In particular, various gene-silencing platforms, including double-stranded RNA interference^57^ and clustered regularly interspaced short palindromic repeats technology^58^, could provide unique opportunities to systematically investigate essential orthologues as intervention targets in *L. cuprina* and to explore in-depth the functions of orphan genes/gene products in this fly. Understanding the functions of essential genes, particularly those involved in reproduction, could pave the way to the development of a sterile insect technique^59^,^60^ for the control of *L. cuprina*, a proposal supported by the success in eradicating the flesh-eating blowfly *Cochliomyia hominivorax* (New World screwworm) from the USA, Central America and some other regions of the world^61^. Clearly, we are now at a point of being able to use the present *L. cuprina* genome and transcriptome resources to address key biological questions, and to facilitate the development of improved tools for blowfly prevention and control in the future. These resources will also support comparative investigations of a range of parasitic dipterans.

## Methods

### Blowfly inbreeding and propagation

A laboratory strain of *L. cuprina* (designated LS)^62^ was maintained for more than 20 years in the laboratory of P.J.J. using an established culture method^63^, employing bovine liver as a medium for ovipositing and larval rearing. Originally, this strain was isolated from the Australian Capital Territory before the use of organophosphate (OP) insecticides and has since had no exposure to insecticides. For this study, five lines were established and inbred for six generations to reduce genetic variability. In each generation, mating pairs of adult *L. cuprina* from each line were kept at 28 °C and 80% relative humidity in separate cages. Each pair was given water and cubed sugar *ad libitum*, and provided with bovine liver on days 1, 2 and 4, to mature ovaries and stimulate ovipositing. The five largest egg masses from each line were selected, and the resultant larvae reared to adulthood on liver within fly-proof containers, with a bed of sand for pupariation and next-generation emergence. A similar procedure was used for producing successive generations, with 8–10 mating pairs (depending on availability) selected from adults emerging from each egg mass (*n*=50 pairs) until the fifth or sixth generation.

### Genomic sequencing and assembly

*L. cuprina* is one of the 30 species whose genome has been sequenced as a part of the pilot project to sequence 5000 arthropod genomes (i5k)^9^ at the Baylor College of Medicine Human Genome Sequencing Center. In the i5k programme, an enhanced Illumina-ALLPATHS-LG sequencing and assembly strategy has been develop to allow the genomes of multiple species to be sequenced in parallel at substantially reduced cost. For the sequencing of the *L. cuprina* genome, we isolated high molecular weight genomic DNA from individuals of each of the mixed larval stages and adults (both sexes) using an established protocol^64^. We constructed and then sequenced four genomic DNA libraries of nominal insert sizes of 180 bp, 500 bp, 3 kb and 8 kb at coverages of 83.6-, 36.5-, 75.1-, 31.1-times, respectively (assuming a genome size of 470 Mb). To construct the 180 and 500-bp libraries, we used a gel-excision, paired-end (PE) library protocol. In brief, 1 μg of genomic DNA was sheared using a Covaris S-2 system (Covaris Inc., Woburn, MA) using the 180- or 500-bp programme. Sheared DNA fragments were purified with beads (Agencourt AMPure XP system, Beckman Coulter), end-repaired, dA-tailed and ligated to universal adapters (Illumina). Following ligation, DNA fragments were further size-selected on agarose gel and then PCR-amplified for six to eight cycles using the primers P1 and Index (Illumina) employing Phusion High-Fidelity PCR Master Mix (New England Biolabs). The final library was purified using beads (Agencourt AMPure XP) and assessed for quality using an Agilent Bioanalyzer 2100 (DNA 7500 kit), determining library quantity and fragment size distribution before sequencing.

Long mate pair libraries, with insert sizes of 3 kb and 8 kb, were constructed individually according to the manufacturer's protocol (Illumina; Mate Pair Library v2 Sample Preparation Guide art. # 15001464 Rev—pilot release). In brief, an amount of 5 μg (for 2 and 3-kb gap size library) or 10 μg (8–10-kb gap size library) of genomic DNA was sheared to the desired size fragments by Hydroshear (Digilab, Marlborough, MA), and then end-repaired and biotinylated. Fragment sizes of 1.8–2.5 kb (2 kb), 3–3.7 kb (3 kb) or 8–10 kb (8 kb) were purified from a 1% (w/v) low-melting point agarose gel and then circularized by blunt-end ligation. These size-selected, circular DNA fragments were then sheared to 400 bp (Covaris S-2), purified using Dynabeads (M-280 Streptavidin Magnetic Beads), end-repaired, dA-tailed and ligated to PE sequencing adapters (Illumina). DNA fragments with adapters on both ends were amplified for 12–15 cycles with primers P1 and Index (Illumina). Amplified DNA fragments were purified with beads (Agencourt AMPure XP). Quantification and size distribution of the final library were determined before sequencing. Sequencing was performed in HiSeq2000 machines (Illumina), generating 100 bp PE reads. Reads were assembled using ALLPATHS-LG (v44620; http://www.broadinstitute.org/software/allpaths-lg/blog/), and then scaffolded and gap-filled using the in-house tools Atlas-Link v.1.0 (https://www.hgsc.bcm.edu/software/atlas-link) and Atlas gap-fill v.2.2 (https://www.hgsc.bcm.edu/software/atlas-gapfill).

### RNA sequencing and assembly

Total RNAs were isolated from adult females (*n*=3), adult males (*n*=8) and mixed larval stages consisting of equal weights of live embryonated eggs (*n*=800), first instar larvae (*n*=800), third instar larvae (*n*=3) and pupae (*n*=3) using Trizol (Invitrogen Inc., USA), treated with TURBO DNase (Ambion Inc., USA) and stored at −80 °C. RNA yields were quantitated spectrophotometrically (NanoDrop 1000; Nano-Drop/Thermo Scientific Inc., USA). RNA integrity was verified using a BioAnalyzer 2100. Following RNA-seq^65^, the sequence-reads derived from individual libraries (representing mixed larval stages, adult females and adult males, respectively) were assessed for quality, and adaptors removed using the programme SeqPrep (https://github.com/jstjohn/SeqPrep). Reads were error-corrected using the algorithms Quake and KmerFreq within the programme SOAPdenovo (http://soap.genomics.org.cn/soapdenovo.html). Extraneous sequences of mammalian, bacterial, mycotic, protistan or plant origin were removed. Subsequently, RNA-seq data for all larval stages and both sexes were assembled *de novo* using the programmes Velvet and Oases (http://www.ebi.ac.uk/~zerbino/velvet/; https://www.ebi.ac.uk/~zerbino/oases/). Non-redundant transcripts were first used to train the *de novo* gene prediction programmes SNAP (http://korflab.ucdavis.edu/software.html) and AUGUSTUS (http://bioinf.uni-greifswald.de/augustus/). Transcripts were then used to assist the evidence-based prediction of the non-redundant gene set for *L. cuprina*.

### Identification and annotation

Genomic repeats specific to *L. cuprina* were modelled using the programme RepeatModeler (http://www.repeatmasker.org/RepeatModeler.html) by merging repeat predictions using RECON (http://selab.janelia.org/recon.html) and RepeatScout (http://bix.ucsd.edu/repeatscout/). Repeats were identified by RepeatMasker Open (http://www.repeatmasker.org) by comparison with modelled repeats (via RepeatModeler) and known repeats in Repbase (v.17.02; http://www.girinst.org/repbase/). The protein-coding gene set of *L. cuprina* was inferred using an integrative approach, employing all transcriptomic data for larval stages (mixed) and adults (both sexes). First, all contigs representing the combined transcriptome for *L. cuprina* were processed using the programme BLAT (https://genome.ucsc.edu/cgi-bin/hgBlat?command=start) and filtered for full-length open reading frames (ORFs), ensuring the validity of splice sites. ORFs were then used to train the *de novo* gene prediction programmes SNAP (http://korflab.ucdavis.edu/software.html) and AUGUSTUS (http://bioinf.uni-greifswald.de/augustus/) by producing a hidden Markov model (HMM) for each programme. The same ORFs were also entered (as an expressed sequence tag input) into the programme MAKER2 (http://www.yandell-lab.org/software/maker.html) to provide evidence for gene transcription. In addition, all quality-filtered reads representing the combined transcriptome were subjected to analysis employing the programmes TopHat (http://ccb.jhu.edu/software/tophat/index.shtml) and Cufflinks (http://cole-trapnell-lab.github.io/cufflinks/), to provide additional information on transcripts and on exon–intron boundaries in the form of a Generic Feature Format (GFF) file. GeneMark *de novo* gene predictions (http://exon.gatech.edu/GeneMark/), HMMs, the expressed sequence tag input and the GFF file were subjected to analysis using MAKER2 to provide a consensus set of genes for *L. cuprina*. Genes inferred to encode peptides of ≥30 amino-acids in length were preserved. To remove extraneous sequences of mammalian, bacterial, mycotic, protistan and/or plant origin(s), scaffolds were broken into contigs at points of indeterminate sequence (Ns). For individual contigs, GC content and average read depth were measured and plotted; then, clusters of contigs with high GC content and low read depth were quarantined, following the verification (via BLASTn) of the origin(s) of extraneous sequences. After this filtering step, genes predicted *de novo* (encoding ≥150 a.a.) by Annotation Edit Distance (AED=1)^66^ were preserved, resulting in the final gene set for *L. cuprina*. Predicted genes were represented by their coding and inferred amino-acid sequences.

### Functional annotation of all predicted protein sequences

First, conserved protein domains of individual inferred amino-acid sequences were identified using the programmes InterProScan 5 and InterPro 44.0 (http://www.ebi.ac.uk/Tools/pfa/iprscan5/; http://www.ebi.ac.uk/interpro/), employing the default settings. Second, amino-acid sequences were subjected to BLASTp (E-value cutoff ≤10^−5^) against proteins in the following databases: FlyBase (*Drosophila melanogaster*, *D. mojavensis*, *D. grimshawi*, *D. pseudoobscura*, *D. virilis* and *D. willistoni*; http://flybase.org), VectorBase (*Aedes aegypti*, *Anopheles gambiae* and *Musca domestica*; https://www.vectorbase.org), Ensembl Genomes (*Megaselia scalaris*; http://www.ensembl.org/index.html), UniProtKB/Swiss-Prot (http://web.expasy.org/docs/swiss-prot_guideline.html), KEGG (release 58; http://www.genome.jp/kegg/) and NCBI protein nr (release September 2013; http://www.ncbi.nlm.nih.gov/).

Individual protein-encoding genes were verified using known KEGG orthology terms by BLASTp analysis (E-value cutoff ≤10^−5^). Homologues were clustered to known protein families using the KEGG BRITE hierarchy employing a custom script. Key protein groups, including peptidases, kinases, phosphatases, GTPases, GPCRs, channels, transporters and TFs, were inferred. ES proteins were initially predicted using the programmes Phobius (http://phobius.sbc.su.se), SignalP v.4.0 (http://www.cbs.dtu.dk/services/SignalP/) and TMHMM v.2.0c (http://www.cbs.dtu.dk/services/TMHMM/) and then inferred to be localized to extracellular space and/or lysosomes (sensitivity: >0.5) employing the programme MultiLoc (http://abi.inf.uni-tuebingen.de/Services/MultiLoc). In the final annotation, predicted proteins were classified according to their conserved InterProScan domains and then based on their homology matches (E-value cutoff ≤10^−5^) to proteins in at least one of six additional databases: (i) KEGG, (ii) FlyBase (*D. melanogaster* and related species), (iii) VectorBase (*M. domestica*), (iv) KinBase using the programme KINANNOTE (http://sourceforge.net/projects/kinannote/), (v) UniProtKB/Swiss-Prot and (vi) UniProtKB/TrEMBL (November 2014). The final, annotated protein-coding gene set for *L. cuprina* is accessible at NCBI in nucleotide and amino-acid formats (Accession code JRES01000000).

### Orthology comparisons

The 14,554 genes of *L. cuprina* were mapped (at the amino-acid level) to orthologous clusters for dipteran flies available via the database OrthoDB8 (http://filemare.com/en-au/browse/cegg.unige.ch/OrthoDB8) using Smith–Waterman database searches with intersequence SIMD parallelization (SWIPE; http://dna.uio.no/swipe/). The resultant clusters were parsed using custom Perl scripts to obtain the numbers of orthologues in *L. cuprina*, *D. melanogaster*, *G. morsitans* and *M. domestica*.

### Synteny

Employing the programme Circos (http://circos.ca), synteny was assessed for the three longest scaffolds (>3.5 million bp) of the *L. cuprina* genome by individually mapping (in a pairwise manner) SCOs (OrthoMCL; http://www.orthomcl.org/orthomcl/), at the amino-acid level, to regions in the genomes of *D. melanogaster*, *G. morsitans* and *M. domestica*. For a given pairwise comparison, a syntenic block of SCOs (*n*≥5) was defined as a set of adjacent genes on a reference scaffold mapping in the same order and orientation to homologous genes on the scaffold being compared (for example, *L. cuprina* *versus* *D. melanogaster*).

### Transcription analysis

Differentially transcribed genes were identified using the programmes RSEM (RNA-seq by expectation maximization; http://deweylab.biostat.wisc.edu/rsem/) and EBSeq (empirical Bayes modelling; https://www.biostat.wisc.edu/~kendzior/EBSEQ/). First, paired-end RNA-seq data representing mixed larval stages (pooled), female adults and male adults of *L. cuprina* were all mapped separately to predicted coding regions (GFF format) in paired-end mode using RSEM (incorporating median normalization) to infer normalized transcript abundance (‘expected counts') for each stage (http://deweylab.biostat.wisc.edu/rsem/). Expected transcript counts were then submitted to EBSeq to generate posterior probabilities of differential transcription among larvae, males and females (https://www.biostat.wisc.edu/~kendzior/EBSEQ/). To minimize false discovery, genes with a posterior probability of differential transcription of 1 and at least 10 expected read counts per gene for at least one stage or sex were considered as differentially transcribed.

### Structural analysis of selected genes

Full-length sequences of five protein-encoding genes (GI: 2894628 for *Ace*; GI: 2565319 for *Rdl*; GI: 1336080 for *Rop1* (*Lc*aE7); GI: 1389670 for *Scl*; GI: KP260561 for *Lc*α6) known or proposed to be involved in particular insecticide resistances in *L. cuprina*^4^ were retrieved from GenBank. Corresponding genomic scaffold(s) were identified using the programme BLASTn. Each coding sequence was aligned to its respective genomic scaffold(s) in Sequencher v.5.2.4 (Gene Codes Corporation; http://www.genecodes.com) using the Large Gap assembly algorithm. PE read data from the 500 bp genomic library were used when multiple scaffolds constituted a gene (for example, scaffold nos. 379, 4253 and 792 for *Lc*α6). Intronic regions were confirmed using transcriptomic (RNA-seq read) data, and intron–exon junctions were confirmed by manual inspection. If required, a reference-guided BWA-MEM alignment (http://www.genecodes.com) was performed to verify the presence or absence of exons in scaffolds and the draft genome assembly.

### Cloning of *Lc*α6

The full-length coding region of the *Lc*α6 gene was PCR-amplified using oligonucleotide primers LucycloneF (5′-GCTGCATTTTTGCTGCATTA-3′) and LucycloneR (5′-TATCGCCAGTTTTGCAAGTG-3′) with a high-fidelity *Taq* polymerase (Expand High Fidelity^PLUS^, Roche) from cDNA (Superscript III, Invitrogen), synthesized from RNA isolated (TRIZol) from *L. cuprina* adult heads. The product was cloned into the p-GEM-T-Easy vector (Promega), sequenced (Macrogen) and then shuttled into the *Not*I site of plasmid pUASTattB (Promega) to produce the construct designated UAS-*Lc*α6.

### Heterologous expression of *Lc*α6 in *D. melanogaster*

Flies homozygous for *dα6*^W337*^ or *dα6*^*nx*^ are 61-fold and at least 1,176-fold more resistant to spinosad compared with the spinosad-susceptible parental line Armenia^14^, an isofemale line derived from the *Drosophila* Genetic Resource Centre stock #103394 (ref. 44). To allow expression in the *dα6*^W337*^ or *dα6*^*nx*^ spinosad-resistant background, the P{w+mW.hs=GawB}elav^C155^ (Bloomington *Drosophila* Stock Centre; BL458) GAL4 driver line of *D. melanogaster*^67^ was crossed separately into a background of *dα6*^W337*^ or *dα6*^*nx*^ spinosad-resistant alleles (chromosome 2) and made homozygous to create elav>GAL4 driver lines for expression experiments. The UAS-*D*α6 line has been reported previously^44^. The landing-site strain expressing the ΦC31-integrase (ΦX-86Fb)^46^ was provided by the Basler Laboratory, University of Zurich, with the second chromosome pair substituted with chromosomes carrying a resistant allele. The fly line with UAS-*Lc*α6 integrated on the third chromosome was created by microinjection into ΦX; *dα6*^W337*^; 86Fb or ΦX; *dα6*^*nx*^; 86Fb lines^44^. The spinosad bioassay for survival to eclosion was performed on standard culture medium^68^, and experimental data were corrected for control mortality using Abbott's formula, adapted for the calculation of 95% confidence intervals^69^.

### Additional analyses

Data analysis was conducted in a Unix environment or Microsoft Excel 2007 using standard commands. Bioinformatic scripts required to facilitate data analysis were designed using mainly the Python 2.6 scripting language and are available via http://research.vet.unimelb.edu.au/gasserlab/.

## Additional information

**Accession codes:** This whole genome shotgun project is available from the DDBJ/EMBL/GenBank databases under the accession JRES00000000 (version JRES01000000). Raw sequences have been deposited in the NCBI short read archive (SRA) under accession code SRS579209. The *L. cuprina* genome sequence is available from NCBI under BioProject accession codes PRJNA248412 and PRJNA203545, as well as BioSample accession codes SAMN02798242, SAMN02947403 to SAMN02947405 and SAMN2422564.

**How to cite this article:** Anstead, C. A. *et al.* *Lucilia cuprina* genome unlocks parasitic fly biology to underpin future interventions. *Nat. Commun.* 6:7344 doi: 10.1038/ncomms8344 (2015).

## Supplementary Material

Supplementary Figures Supplementary Figures 1-3

Supplementary Data 1 Information on genomic DNA sequence and RNA-seq data generated for *Lucilia cuprina*. Core eukaryotic genes are also listed.

Supplementary Data 2 A summary of repeats in the draft genome of *Lucilia cuprina*.

Supplementary Data 3 Proteins encoded in the *Lucilia cuprina*.genome and associated with biological (KEGG) pathways.

Supplementary Data 4 Peptidases encoded in the *Lucilia cuprina*.genome.

Supplementary Data 5 Kinases encoded in the *Lucilia cuprina*.genome.

Supplementary Data 6 Phosphatases encoded in the *Lucilia cuprina*.genome.

Supplementary Data 7 GTPases encoded in the *Lucilia cuprina*.genome.

Supplementary Data 8 G protein-coupled receptors (GPCRs) encoded in the *Lucilia cuprina*.genome.

Supplementary Data 9 Ion channels encoded in the *Lucilia cuprina*.genome.

Supplementary Data 10 Transporters encoded in the *Lucilia cuprina*.genome.

Supplementary Data 11 Transcripts enriched in female adults of *Lucilia cuprina*.

Supplementary Data 12 Transcripts enriched in male adults of *Lucilia cuprina*.

Supplementary Data 13 Transcripts enriched in mixed larvae of *Lucilia cuprina*

Supplementary Data 14 Secretome predicted from the *Lucilia cuprina* genome.

Supplementary Data 15 *Lucilia cuprina* orthologs of essential genes in Drosophila melanogaster

Supplementary Data 16 Genes/proteins predicted to be druggable in *Lucilia cuprina*.

## Figures and Tables

**Table 1:** Features of the draft genome of *Lucilia cuprina*.

**Description**	
Total number of base pairs (bp) within assembled scaffolds	458,190,778
Total number of scaffolds; contigs	4,436; 74,043
N50 length in bp; total number >N50 in length	744,413; 165
N90 length in bp; total number >N90 in length	126,471; 736
GC content of the whole genome (%)	29.3
Repetitive sequences (%)	57.8
Proportion of the genome that is coding (exonic; incl. introns; in %)	6.2; 34.7
Number of putative coding genes	14,554
Gene size (mean; bp)	12,197
Average coding domain length (mean; bp)	1,455
Average exon number per gene (mean)	4.50
Gene exon length (mean; bp)	432
Gene intron length (mean; bp)	2,560
GC content in coding regions (%)	39.2

**Table 2:** Key protein groups encoded in the *Lucilia cuprina* genome.

**Protein group**	Numbers predicted*
Transcription factors	446
Transporters	367
Peptidases	260
Excretory/secretory proteins	234
Phosphatases	199
G protein-coupled receptors	197
Kinases	167
Ion channel proteins	136
GTPases	92
Peptidase inhibitors	34
Major sperm proteins	34
Vitellogenins	20

^*^Some predicted proteins belong to multiple categories.

**Table 3:** Isoform and RNA editing status of α6 expression constructs.

**Construct**	**Alternate exons**	A-to-I RNA-edited sites*
UAS-*D*α6	3b, 8b	4, 5 and 6
UAS-*Lc*α6†	3b, 8a	4, 5, 6 and 7

^*^RNA editing sites numbered according to Perry *et al.*^44^

^†^GenBank accession no. KP260561.
