# Supplementary material for: Lucilia cuprina genome unlocks parasitic fly biology to underpin future interventions
Source: Nat Commun. 2015 Jun 25;6:7344. doi: 10.1038/ncomms8344 (PMC4491171; doi:10.1038/ncomms8344)
Supplement: Supplementary Figures — 1-3 [file ncomms8344-s1.pdf]

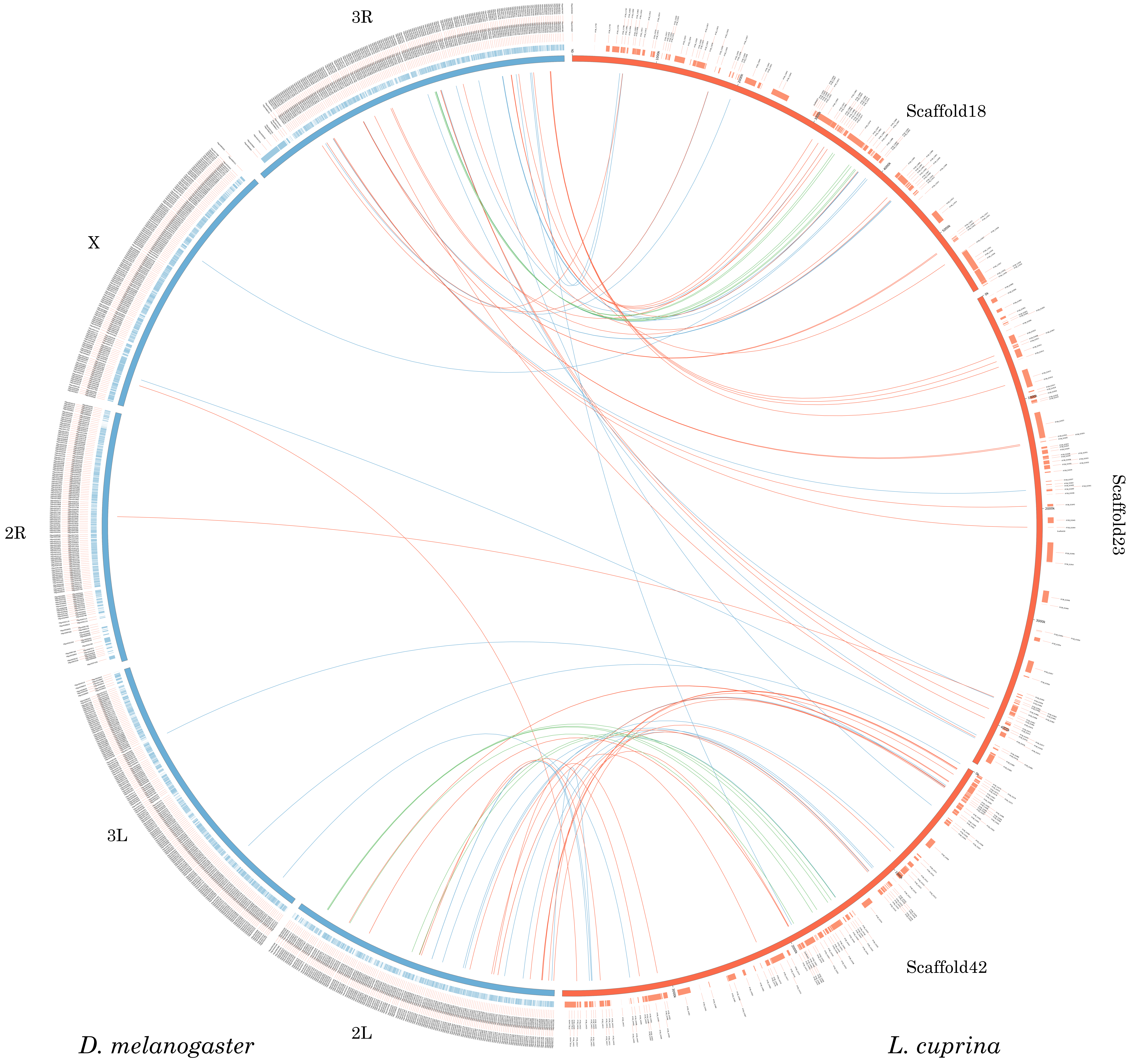

Supplementary Figure 1 | Syntenic comparison between *Lucilia cuprina* and *Drosophila melanogaster*. Mapping of homologous single-copy orthologs (SCOs) located on three of the longest scaffolds (nos. 18, 23 and 42; > 3.5 million bp) found in the *L. cuprina* genome to *D. melanogaster* chromosomes. Blue lines map SCOs found on the same strand, whereas red lines map SCOs to regions on opposite strands. Green lines correspond to blocks of > 5 adjacent SCOs that map to the same scaffold region. Mapping indicates only small numbers of scaffolds sharing blocks of > 5 SCOs (n = 2; scaffolds nos. 23 and 42) in the comparison.

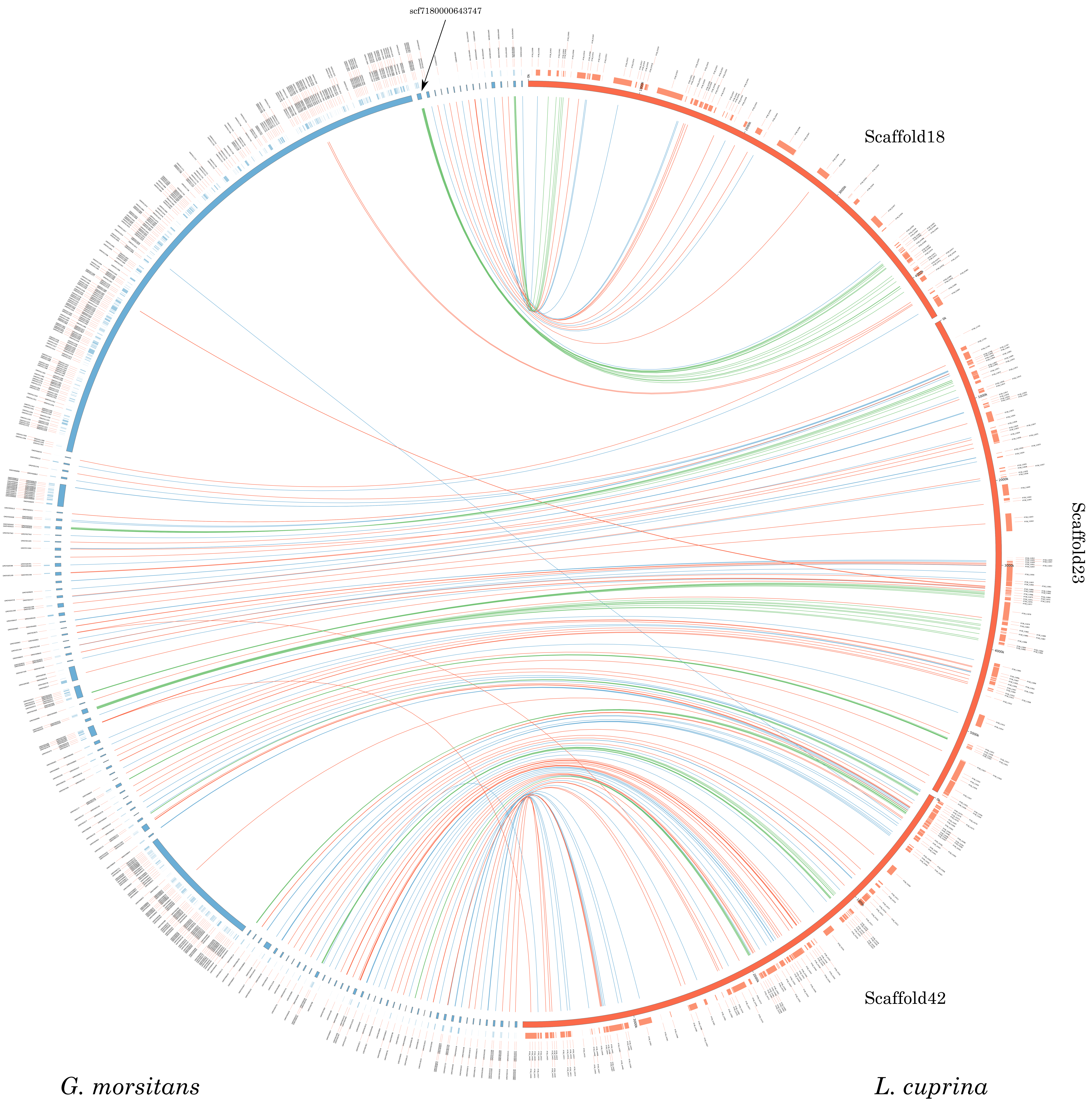

Supplementary Figure 2 | Syntenic comparison between *Lucilia cuprina* and *Glossina morsitans*. Mapping of homologous single-copy orthologs (SCOs) located on three of the longest scaffolds (nos. 18, 23 and 42; > 3.5 million bp) found in the *Lucilia cuprina* genome to those in *Glossina morsitans*. Blue lines map SCOs found on the same strand, whereas red lines map SCOs to regions on opposite strands. Green lines correspond to blocks of >5 adjacent SCOs that map to the same scaffold region. Mapping indicates only small numbers of scaffolds sharing blocks of > 5 SCOs (n = 5; scaffolds nos. 18, 23 and 42) in the comparison; the second largest block of SCOs (n = 13) were found between *L. cuprina* (scaffold no. 18) and *G. morsitans* (scaf7180000643747).

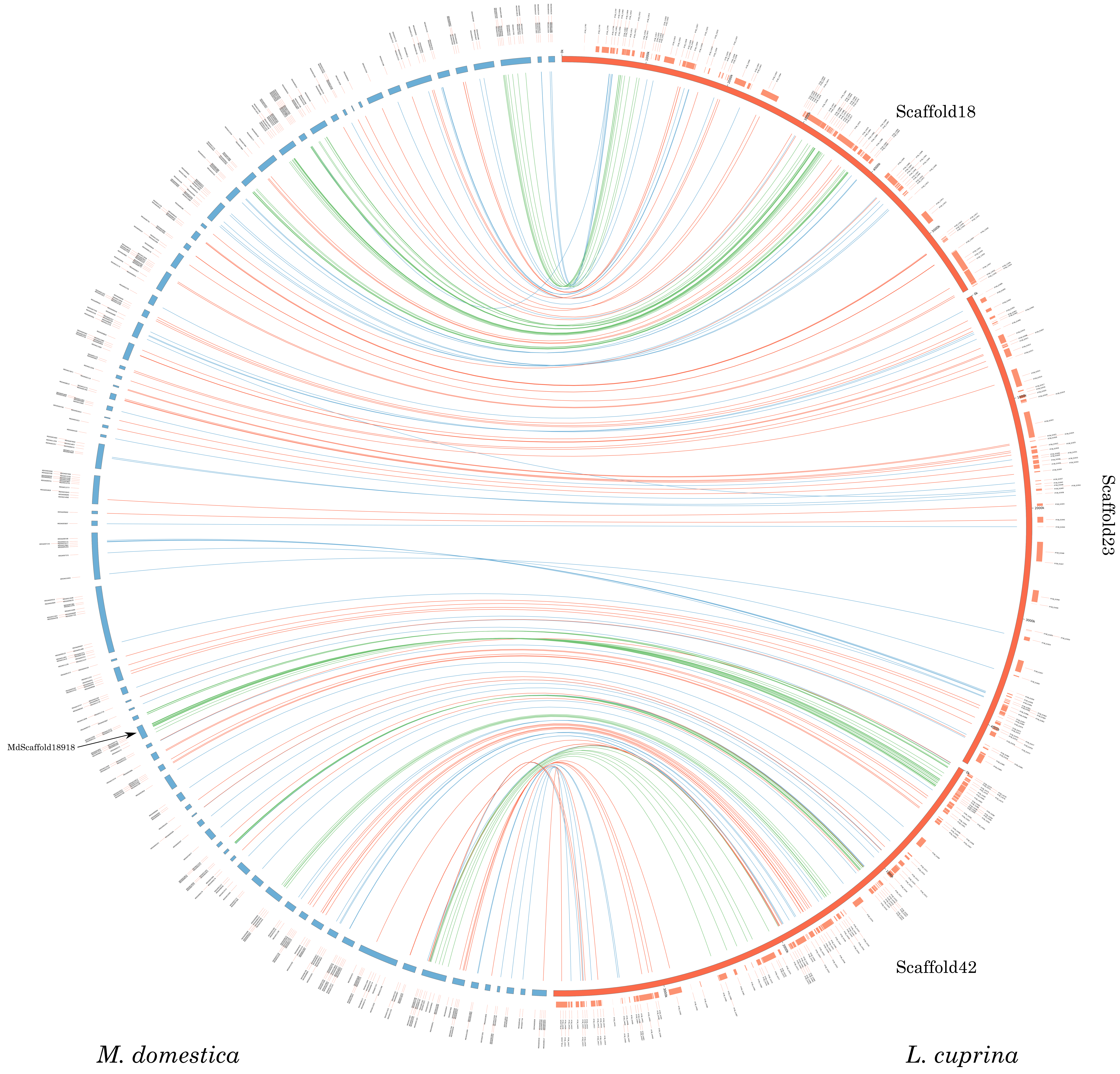

Supplementary Figure 3 | Syntenic comparison between *Lucilia cuprina* and *Musca domestica*. Mapping of homologous single-copy orthologs (SCOs) located on three of the longest scaffolds (nos. 18, 23 and 42; > 3.5 million bp) found in the *L. cuprina* genome to those in *M. domestica*. Blue lines map SCOs found on the same strand, whereas red lines map SCOs to regions on opposite strands. Green lines correspond to blocks of >5 adjacent SCOs mapping to the same scaffold region. Mapping indicates only small numbers of scaffolds sharing blocks of > 5 SCOs (n = 6; scaffold nos. 23 and 42) in the comparison; the largest block of SCOs (n = 15) was found between *L. cuprina* (scaffold no. 42) and *M. domestica* (MdScaffold18918).
